# Medico-Chirurgical Transactions. Vol. XX

**Published:** 1837-10-01

**Authors:** 


I. Medico-Chirurgical Transactions. Published by the Royal Medical and Chirurgical Society of London, for 1836. Volume the Twentieth.

II. The Transactions of the Provincial Medical and Surgical Association. Volume V.

III. Elements of Medicine. Vol. I. On Morbid Poisons. By *Robert Williams*, M.D. &c. &c.

The major portion of the Papers in the former, and a considerable part of those in the latter of these three volumes, have been already noticed in previous numbers of this Journal. It only remains for us to complete our account of them.

1. Observations on Vascular Appearances of Mucous and Serous Membranes, as indicative of Inflammation. By John Yelloly, M.D. F.R.S. &c. &c.

Dr. Yelloly observes, with truth, that nothing is more important for the successful prosecution of morbid anatomy, than the habit of accurately connecting the phenomena which present themselves after death, with the symptoms which occur during life; and of carefully distinguishing between the varieties of form and appearance which exist in healthy parts, and such as are the result of derangement or disease. He goes on to remark how necessary very careful attention and discrimination are, and how little disposed the best qualified pathologist sometimes feels himself to speak with decision on the nature of some appearances which come before him. Nothing can be more correct than are these sentiments, and all who are much acquainted with morbid anatomy, must have had sufficient opportunities of observing the confidence and decision of ill-informed persons on points of pathology which are really involved in uncertainty and doubt.

This is particularly the case with inflammation, a process so important that we ought to be thoroughly acquainted with it, and not always so distinct in its existence or its consequences as to permit us to speak positively with respect to either.

The characters of external inflammation are too well known to require repetition. It is known, too, because it is an object of sense, that death makes important alterations in the appearances which such inflammation presents. The redness goes off, except where effusion has taken place, and the remains of it are seen; while the tumor sinks, except in as far as any deposition in the cellular membrane has taken place, and may render it permanent. The blood, in fact, leaves, more or less completely, the arteries and veins, and all the phenomena which depended on its presence in them necessarily disappear in the same ratio.

" But,' says Dr. Yelloly, ' in the internal organs, and more particularly the abdominal viscera, various modifications take place, from the peculiar situation which these parts occupy in the animal body. Being nearly connected with a double series of venous structure, that of the liver, and that of the heart, in which a considerable portion of the blood is concentered after death, a retarding or obstructing cause operates on the veins which belong to them, and which, added to the softness of texture to which I have already alluded, very generally produces more or less fulness of the vessels, and thus an appearance of more or less vascularity, both on the external and internal surfaces of the chylopoietic viscera." 10.

It follows, as a matter of course, *that* vascular fulness in the dead body is delusive—*that* it is not present, when undoubted inflammation existed during life upon the surface—*that* it is present in the interior from the operation of circumstances which did not obtain before death—and *that* a knowledge and a careful consideration of these disturbing circumstances is indispensable, to enable the pathologist to decide what appearances are and what are not inflammatory. A florid state of vessels after death, for example, has often been regarded as a proof of arterial congestion, yet it merely arises from the arterial character of the blood remaining in the veins, for some time after its transmission from their continuous arterial capillaries.

The profession must feel indebted to any one who puts it in possession of facts which will assist it in the discrimination of genuine inflammatory appearances in the interior of the body. It has been Dr. Yelloly's aim to offer that assistance, and not only on this, but on a previous occasion, he has laid the results of many careful investigations before the medical public.

In the fourth volume of the Medico-Chirurgical Transactions, published in 1813, will be found a paper by our author, on the Vascular Appearances of the Human Stomach, which are often mistaken for inflammation of that organ. We shall recapitulate what he laboured to establish in that paper, for two reasons: because, the majority, perhaps, of our readers are unacquainted with them, and because the deductions are not only highly important in themselves, but are so interwoven with the observations in the present paper, that it would be unnatural and mischievous to separate them. Dr. Yelloly, then, in his first paper, endeavoured to shew.—

1st. That appearances of vascular fulness in the villous coat of the stomach, whether florid or dark-coloured, in distinct vessels, or in extravasations of different sizes, are not to be regarded as unequivocal marks of disease; inasmuch as they occur in every variety of degree and character, under every circumstance of previous indisposition, and in situations where the most healthy aspect of the organ is to be expected.

2d. That from the circumstance of such appearances being in many cases not preceded by symptoms of inflammation, many authors of great eminence were led to the conclusion, that gastritis might exist in the living body, without the indications of either pain or pyrexia; that it was a very common and frequent complaint, even terminating occasionally in gangrene, though there were no indications during life of its existence: and that the absence of the usual symptoms of inflammation of the stomach, when unequivocal marks of such inflammation were supposed to be found on dissection, could not but be regarded as a very remarkable, and puzzling circumstance.

3d. That the same misconceptions attached to intestinal vascularity; and that MORGAGNI himself, in order to get quit of the difficulty, was in some cases disposed to attribute the absence of pain, in inflammation of the bowels, to a paralytic affection, which took off the sensibility of the parts; but that, as this was a circumstance which could but seldom occur, he came to the general conclusion, that pain and fever were not necessary for the existence of enteritis. Haller, too, was induced to infer, from so uniformly finding vascularity in his inspections, that inflammation of the bowels was almost constant in every kind of fever, and was frequent in every other complaint.

4th. That the supposition of a necessary connection between vascularity and inflammation, has led to erroneous conclusions in many cases of sudden death, where the effects of putrefaction, and the spontaneous changes which the loss of vitality produces on the human body, were misunderstood, and sometimes confounded with the proper and necessary operation of poisons.

5th. That some diseases of a very obscure nature, as hydrophobia, have been considered as examples of gastritis, and plans of extensive depletion adopted for their treatment, merely from the villous coat of the stomach exhibiting appearances of vascularity, though there was no palpable difference between such vascularity, and that which is ordinarily seen where there had been no affection of the stomach during life.

6th. That such vascularity is entirely venous, though sometimes florid, and sometimes of a dark red; that it depends on a power capable of being exercised on the artery itself at the close of life, which carries on the blood to the veins, after the further supply of fresh blood from the heart is stopped; and that the branched or stellated form of vessels, under which the vascularity usually appears, is capable of being imitated, whether by injection of the veins with fine injection, or by forcing back with the finger, or the back of a scalpel, the blood from the larger branches of veins into the smaller.

7th. That vascularity soon becomes diffused redness, by transudation through the coats of the containing vessels, just as happens with the bile in the gall-bladder, or in the speedy production of a blue colour, when the mesenteric arteries are injected with a solution of prussiate of potash, and the veins with that of green sulphate of iron.

8th. That digestion of the coats of the stomach after death, is by no means a common occurrence; inasmuch as extravasation does not take place in the great end of the stomach, however thin its coats may be, when fine injection is thrown into it, either by the superior coronary, or the splenic artery, as would be the case, if there were usually an erosion of the ends of vessels produced after death, by the action of the gastric juice, according to the opinion of Mr. Hunter.

9th. That it is very difficult to ascertain, merely from the aspect of the vessels of a dead part, that they had been affected with inflammation during life, unless for the occurrence of unequivocal results of inflammatory action.

The preceding views are especially applicable to mucous surfaces—to the mucous membrane of the stomach in particular: then, to that of the intestines: then, to that of the trachea. It is the laxity of the mucous membrane, which renders it so liable to vascular turgescence after death.

They apply, with modifications, to the serous membranes. These, in health, exhibit no vessels which carry red blood; when inflamed, effusion of coagulable lymph very quickly occurs.

On the surface of the brain and cerebellum vascular appearances often occur, which, like those already mentioned, are principally the result of venous accumulation after death.

The spinal marrow is so seldom examined, that its healthy and morbid appearances are not, in general, accurately known. In order to ascertain the vascular appearances of the spinal marrow, uninfluenced by any disturbing cause except a particular mode of death, Dr. Yelloly examined it in three executed criminals. He subjoins a coloured drawing taken from one, and he adds, that—a florid vascularity, communicating a scarlet line to the whole column, is nothing more than venous turgescence, accompanied, as in the case of the stomach, depicted in his former paper, with slight extravasation.

Dr. Yelloly proceeds to examine and to criticise the foundations of the doctrines of Broussais and of Tommasini, the former of whom attributes general fever to inflammation of the mucous membrane of the stomach and intestines—while the latter regards it as essentially inflammation of the brain. The appearances on which these pathologists rely, as proofs of the correctness of their views, consisting essentially in increase of vascularity in the parts concerned, rather than in the presence of the undoubted effects of inflammation, we may readily conceive how delusive they may be. It is unnecessary for us to go into the question, for the hypotheses of Broussais and of Tommasini have been long regarded as fallacious. Morbid anatomy, the history of disease, and the effects of remedies, have all combined to shew how hollow such exclusive theories are.

Quitting Broussais and Tommasini, Dr. Yelloly turns to M. Andral, a more exact, a less speculative, and, consequently, a more philosophical pathologist. In his earlier writings, M. Andral did not escape the error into which the former gentleman had fallen, and the appearances of inflammation and congestion were far from being distinguished with precision. But in his Précis d'Anatomie Pathologique, M. Andral has drawn the line more carefully and more accurately between the two conditions. It is well known that M. Andral has endeavoured to banish the term inflammation, and to substitute for it that of *hyperœmia*. In his nomenclature, inflammation is represented by *hyperémie active* ou *sthenique*—congestions, by *hyperémie passive* ou *asthenique*, when there is a diminished tone in the capillary vessels; *hyperémie mécanique*, when there is an obstacle to venous circulation; and *hyperémie cadaverique*, which occurs after death.

His deductions with respect to the *hyperémie cadaverique* are very similar to those arrived at by Dr. Yelloly in his former paper, both in reference to its cause, the circumstances under which it occurs, and its liability to be mistaken for a morbid appearance. We quote the following extract from Dr. Yelloly's paper, because the criticisms on M. Andral's opinions, are not deficient in pathological value.

"He considers it," says Dr. Yelloly, "as established by his observations, that the hyperemiæ which have come on during life, or after death, are not always to be distinguished from each other by their anatomical characters; and that simple inspection does not, in a great number of cases, suffice to determine the cause of sanguineous congestions found in pathological examinations. The gastro-intestinal membranes, he continues, may be indifferently white or red, according to the circumstances under which death has taken place; but as the circumstances which produce a red colour are most frequent at the close of life, it will oftener happen, that congestion of vessels in the alimentary canal will be found in the dead body, than a want of colour. It does not follow, however, he adds, that a red colour is any proof that disease existed in the alimentary canal during life; inasmuch as an intestine found to be red in the dead body, was not necessarily so during life.

M. Andral attributes the position of intestinal congestion, with Messrs. Trousseau and Rigot, to the mechanical descent of the fluid blood, according to the posture which the body is made to assume after death; and in one case, where the vascularity was in the anterior part of the stomach, owing to the body lying on its back, he was disposed to refer the appearance to *gastritis*, which, with much candour, he states he should not do now. That this cause operates, to a certain extent, in producing the phenomenon in question, particularly when the larger vessels are concerned, is quite certain; but, though I have remarked, that vascularity is found 'principally in the posterior part of the great end of the stomach, and in the lesser curvature,' yet nothing is more common than to see great turgescence of vessels on the upper surface (in relation to the position of the body) both of the intestines and brain; and it occurs everywhere in the minute vessels of the inner membrane of the stomach and bowels, where an attraction is exercised between the coats and their contents, which prevents any material change of position in the latter.

M. Andral is disposed to refer the transudation which takes place through the sides of vessels some time after death, to *putrefaction*. But I see no reason to alter the opinion which I have expressed on this subject, namely, that 'putrefaction will doubtless increase, but it does not seem at all *necessary* to transudation.' The animal fibre has a power to resist the passage of a fluid during life; but very soon after death, and long before putrefaction can be considered as having at all taken place, the power is lost, and various well known phenomena speedily occur in every part of the body, which are dependent on that unknown and inscrutable change, which the loss of vitality has effected in the animal machine. Transudation of bile from the gall-bladder, and of blood into the thoracic or abdominal cavities, are among the commonest examples; and the veins of the stomach and bowels will exhibit the same appearance in a day, or day and a half from death; but in a still shorter time if an inverted vascular stomach is extended upon a flat surface, and kept moist and undisturbed. I may mention too, as an additional proof of the same circumstance, the point mentioned at page 4 of this communication; and may add, that the same will happen in a tied carotid artery or intestine, containing either of the fluids employed, and made to dip into a vessel containing a portion of the other." 22.

There is a point which deserves some notice, because we cannot doubt that inattention or ignorance respecting it has not unfrequently led to error. It is this. The follicles which are sometimes exceedingly distinct in various parts of the stomach may readily be mistaken for minute ulcerations. To avoid that mistake it is necessary, of course, to keep the liability to it in view. Dr. Yelloly suggests, as a mode of determining the nature of the case, the employment of a minute injection. If there was ulceration, the injection would be extravasated. We fear the test is of too difficult application to be generally employed, and that the most practical recommendation is, to use great care in distinguishing the real state of the parts.

Dr. Yelloly alludes to the opinion of Otto, Trousseau, Rigot, and Billard—opinions which correspond more or less exactly with his own, and he adds:—

" It would have saved them, however, much labour, and perhaps, to a certain degree have modified some of their deductions, had they been so far acquainted with our Transactions, as to have known what had been done in this country on the same subject. Life is, indeed, too short, and the difficulties which attend the prosecution of philosophical inquiries too considerable, to afford that there should be no division of labour in science; and that men of talents and energy should occupy themselves in investigations which have already been prosecuted in other quarters, without being aware of what has already been done on the same subject. Science is common property, of which every one may avail himself at his pleasure; but the talents of able men belong also in a certain degree to the public; and it is a species of duty for such as are qualified, by their abilities and industry, to enlarge the boundaries of science, to employ themselves in the most provident manner possible for the common benefit."* 27.

We have quoted this passage, because it represents a fact which should be known. We are amongst the last who are likely to undervalue the labours of the French in science, and, more particularly, in pathology. But their very success has tended to encourage that vanity which is said to be their national characteristic, and to make them undervalue and neglect whatever is not done by themselves. The ignorance of the French, in reference to the state of medicine in this country is preposterous. Whilst they are behind us in all that is practical, with the exception of the mere *performance* of operations, they exhibit, if they do not affect, an utter indifference to our labours and our existing knowledge, and rarely notice either, unless it be to misconceive or to mis-state them.

The consequences are such as might have been expected. Even in pathology, we daily see facts or opinions published as novelties by French writers, when they have been long anticipated here.† In practical medicine and surgery, we see plans of treatment proposed and lauded, which experience in this country has already sanctioned or condemned. We find ideas of disease prevailing undisturbed in France, which science has exploded in England.‡ And, finally, we are visited every now and then, by some French tourist, who, knowing little of our language, nothing of our literature, ignorant of our practice, and incapable of understanding it—pays a hurried visit to a few hospitals, where he sees not much, and comprehends much less—yet, is so charmed with his own sagacity, and so contented with his amount of information, that he publishes on his return to his own country, a full, and of course an accurate account of the medical science and practice of this.§

---

* "Considering the almost national character of the Medico-Chirurgical Transactions, it is very remarkable how little they seem to be known among the best French medical writers, even in reference to some of our most distinguished names. A curious exemplification of this occurs in Cruveilhier, who informs us in the 3d livraison of his Anatomie Pathologique, (pl. 6, p. 10) that he lately made a very convincing experiment ('une experience bien convaincante'), to prove, that disease on one side of the spinal marrow, produces its effect on the same, and not the opposite side of the body. But the point had been examined many years previously by Sir Astley Cooper, at my request, in consequence of the contradictory evidence on the subject; and a similar conclusion, confirming a valuable experiment of Galen, arrived at by direct experiment on a dog, of which an account was published by me in the first volume of the Medico-Chirurgical Transactions, p. 197."

† MM. Bouillaud and Magendie have, on several occasions, been convicted of this neglect of English medical literature.

‡ The French constantly treat chronic inflammation of the testis as cancer. They retain the terms and the ideas of white swelling, &c.

§ A Lyons surgeon lately gave a striking example of the assurance resulting from profound ignorance of all that a traveller, who affects to describe a foreign nation, should know.

### II. On the Effects of Morbid Poisons.

### 1. *On the Treatment of Injuries received in Dissection. By R. A. Stafford, Esq. Surgeon to the St. Marylebone Infirmary.*

The effects and the management of dissection-wounds, possess, of course, a painful interest for ourselves. They are the capitation tax we pay for knowledge, and we are naturally concerned in rendering it as light as possible.

Mr. Stafford fairly states the case, and takes, we think, the most reasonable view of it in the following introduction to his paper.

" Three opinions have prevailed in the profession regarding the nature of the symptoms resulting from these injuries: one, that a peculiar virus is introduced into the system, which so influences the nervous and vascular functions, as to occasion the alarming symptoms: another, that the latter depend on the nature of the part injured, as in the case of puncture of the sheath of a tendon, &c.; and a third, that impaired general health preceding the accident is the cause of the symptoms.

We may admit constitutional predisposition in all cases: but it appears to me that the phenomena can only be satisfactorily accounted for on the first supposition, that of the introduction of an animal poison. For we find the most alarming and rapidly fatal cases to occur where there has been but a slight abrasion or scratch, as well as where a deeper wound has been inflicted by the point of the scissors, the hook, or the scalpel, so as to render questionable the probability of wounding the sheath of a tendon. Further, many cases are recorded in which no abrasion, scratch, incision, or puncture was noticed, or any evidence of such injury found on the most minute inspection of the part; consequently some deleterious poison must have been absorbed by the skin. I shall produce remarkable instances of this mode of receiving the injury. It is also a curious fact, that in most of the cases recorded by Dr. Duncan, Dr. Colles, Mr. Travers and others, and where the symptoms were the most severe, and the result fatal, the wound was generally no larger than a puncture by a needle, or a slight abrasion." 51.

We do not understand how some persons can contend, that the phenomena which result from dissection-wounds are not referable to inoculation with a morbid poison. The evidence is of the same description as that which is received as proving inoculation with other morbid poisons. It is true that there are some differences between the consequences of dissection-wounds and the consequences of other poisoned wounds, but those differences are rather of quality than kind. It must be recollected, that the resemblances between the phenomena occasioned by various morbid poisons are not close, each poison appearing to act by special laws. It is not just, then, to try one by the formula of another, and to insist on slight discrepancies as subversive of their analogy and their relationship.

The strongest argument that has been advanced against the agency of a specific poison in dissection-wounds, is the necessity for a certain constitutional state on the part of the recipient, in order that certain consequences may ensue. It has been argued that these cases usually occur at the termination of a scholastic session, when the constitutional powers of students are impaired.

This argument, however, has been overstrained, for there are, unfortunately, too many instances of individuals dying of dissection-wounds, who, previous to the reception of the injury, were, to all appearance, in good health. And, granting to the argument all the force that is claimed for it, it still goes no farther than to shew, that, for the poison to act seriously upon the system, the latter must be debilitated and unable to resist it. This is quite consistent with what occurs when individuals are exposed to the action of other morbid poisons. Nothing can be more certain than that, cæteris paribus, the more impaired the constitutional powers, the more likely a person is to be affected by a morbid poison, and, when affected, to suffer severely.

But there is another law which interferes with the one we have just stated, and involves the subject in much of the obscurity which unhappily distinguishes it. It is the law of predisposition. The facts on which we ground its existence are ultimate facts, and incapable of explanation. The final cause is obvious—the quomodo unintelligible. We know *that*, of a number of persons exposed, at different times, to the influence of a morbid poison, some will be affected by it at one time, some at another, and the majority not at all—*that* those who resist it upon one occasion, may, under apparently similar circumstances, be incapable of resisting it on another—*that*, of those who are affected by it, some will be so in a slight degree, and others, commonly the minority, in an excessive one—and, finally, *that* appreciable constitutional states will not explain this predisposition or indisposition for the reception of any particular poison.

It is clear that if there were no law of predisposition, either pestilences would never have existed, or they would long ago have depopulated the earth. Why pestilences should come, why morbid poisons should be generated, we do not take on us to say, but we would observe that, the necessity for death being granted, pestilences and morbid poisons would naturally seem to spring from the operation of those causes by which death is effected; and that they are the consequences of such natural causes, is rendered still more probable by their diminution, in consequence of the improved physical and moral habits of nations. Setting aside, then, the question why some should be constitutionally liable to suffer from the operation of morbid poisons, we must be grateful to the great Origin of all things, that the majority of men are not. It is here, as we observed, that the final cause is intelligible, and if there is an interruption to the operation of natural causes, it is for the preservation and not the injury of the animal creation.

Another argument which has been advanced against the supposition of the operation of a specific poison in cases of dissection-wounds, is, the variety of effects to which it must be admitted to give rise. In one person the punctured part will merely fester—in another, inflammation of the absorbents will ensue—in another, there will supervene diffuse cellular inflammation and extensive suppuration—in another, there will be a peculiar train of symptoms rapidly terminating in the destruction of the individual. It has been said that, in persons of bad constitutions, or, sometimes independently of any tangible constitutional vitiation, the simplest injuries will give rise to many of the consequences, which, in cases of dissection-wound, we attribute to the action of a poison.

But it may be observed, that injuries so slight as dissection-wounds almost always are, rarely give rise to any mischief at all, and, even when they do so, occasion no more than inflammation of the absorbents or cutaneous erysipelas. Nothing can be less common than severe diffuse cellular inflammation from a mere prick or scratch of a needle; and no one has seen an ordinary injury of that description succeeded by those strange and fatal symptoms which are too frequent after wounds in dissection. In fact, we may state it as a general proposition that, in ordinary wounds, the effects are more or less in the ratio of the appreciable amount of injury—while in dissection-wounds they are quite out of proportion to it.

It may be urged that this argument by no means proves that the slighter consequences of dissection-wounds are the result of inoculation with a specific poison. That is true, nor have we any means of determining the point. We may infer, if we are inclined, that the slighter and the ordinary as well as the graver and extraordinary sequelæ depend on the same or on a similar cause, the effects of which are modified by disturbing circumstances; but the former supposition does not rest on the same amount of proof or probability as the latter does. We must admit one of three things—*that,* the same poison will, under different circumstances, produce all the different phenomena, which we observe in cases of dissection-wounds; *that,* there are several poisons; or *that,* only the severer cases are to be regarded as the produce of inoculation with a specific poison, the slighter ones being merely instances of the effects of common irritation acting on a peculiar state of constitution. There are difficulties attendant on the reception of each of these hypotheses. But the last, whether strictly true or not, agrees best with practical experience, and best explains the results of treatment.

Having made these few remarks, and endeavoured to clear the way for Mr. Stafford's cases, we shall now proceed to them. He relates six, of which only one proved fatal. We shall notice the more important.

*Case* 1. Dr. Sims dissected a brain on the 1st of March, 1833. He was not aware of having pricked himself in any way.

At 4, a. m. of the 2d, he was awoke by pain in his right fore-finger. He got up and took a five-grain dose of calomel. At 8, a. m., he sent for Mr. Stafford. There was then an elevated spot on the inner surface of the second phalanx, resembling the bite of a gnat, but there could not be discovered in any part of it, not even by a magnifying glass, the slightest trace of a puncture. The finger was extremely painful, and considerably swollen and inflamed. The inflammation of the absorbents extended in two or three distinct lines along the inner side of the fore-arm nearly up to the ulnar side of the elbow-joint. The pulse was hard and at ninety, having that thrilling or jerking stroke which indicates constitutional disturbance; the tongue was coated by a dirty white fur, and the skin was somewhat hotter than natural. Eight leeches were applied to the finger; an evaporating lotion on the fore-arm, and a senna draught taken. From the rapid progress of inflammation of the absorbents, the case put on an increasingly unfavourable aspect. Between twelve and one Dr. Lee called upon him; he was then much worse. The pain in the finger had greatly increased, and the inflammation of the absorbents had extended nearly to the axilla. The pulse was small and irregular, wavering from 120 to 130. There was a peculiar physically anxious expression of the countenance, accompanied with a nervous tremor over the whole frame; the voice was tremulous, and occasionally there was a transient incoherency, which, however, Dr. S. appeared conscious of, and could immediately command. In addition to these symptoms, he suffered a most excruciating pain in the lumbar region, which caused a constant involuntary tremor and twitching of the lower limbs, and more particularly on the right side. In fact, the whole nervous system appeared to have received so great a shock, that the symptoms almost resembled those produced by the bite of a venomous reptile.

The nitrate of silver was rubbed on the arm along the course of the absorbents, and Sir A. Cooper and Mr. Lawrence were called in. The latter only could at first attend, which he did between two and three, p. m. The symptoms had then increased. There was extreme depression of the nervous system, and there were convulsive twitchings of the lower limbs. Twenty more leeches were immediately applied to the finger, and along the course of the inflamed absorbents; the hand and arm were afterwards well fomented. Twelve grains of the compound powder of ipecacuanha were taken, but immediately rejected. A fourth of a grain of the muriate of morphia in solution was therefore substituted. An aperient was also exhibited.

At 8, p. m., the pain and nervous symptoms were somewhat diminished, but the finger was still more swollen and tense, and the palm of the hand and third phalanx were swollen too. The bowels had acted.

A deep longitudinal incision was made along the flexor tendon of the second phalanx, but no pus escaped. To continue the fomentation, and apply twenty more leeches to the hand and absorbents. Repeat the muriate of morphia as often as occasion may require.

March 3d, 4, a. m. After the leeches had been applied and the morphia taken in the evening, Dr. S. dozed for an hour or two. When the muriate of morphia had been taken half an hour, all the symptoms abated. The pulse was reduced from 130 to 100, and beat with a firmer stroke. The tremors were lessened and the pain in the back and hand considerably diminished. The effect of the morphia had now ceased; the pain in the hand and particularly the finger was excessive. The pulse had increased to 140, and wavered irregularly. The tremors and twitchings of the limbs were still more violent than they had been, and the pain in the back excruciating. The depression of the nervous system was even greater than it ever had been; so much so, that unless relief could be obtained, a speedy dissolution might have been expected. Half a grain of the muriate of morphia was immediately given. The effect was remarkable. In five minutes it began to act. The pain gradually diminished, the pulse fell to between 90 and 100, and Dr. S. fell asleep. In two hours, however, the symptoms began to return, and the same dose of morphia was administered with the same good effect. It was ordered to be repeated every second hour, as long as the symptoms demanded it.

8, a. m. The hand is in much less pain, but the swelling on the third phalanx and in the palm has increased. The other symptoms rather mitigated. Twenty leeches to be applied to the absorbents and most painful part of the hand; to continue the morphia, the fomentations, and poultice. He could take nothing but soda and seltzer water, with an occasional cup of tea.

During the day, all the symptoms were alleviated, with the exception of the pain in the hand, which remained the same, and of the swelling of the third phalanx, which was greater and more tense. Twenty leeches to the hand and absorbents. The other measures to be persevered with.

March 4, 8, a. m. Passed a better night, and is improved in all respects. Pulse 98. Bowels have not acted. The third phalanx of the finger is more swollen and tense.

At 3, p. m., the pain, swelling, and tension of the finger, had still farther increased, and slight fluctuation could be felt on the third phalanx. A deep longitudinal incision was made into it, and pus escaped with the blood.

We need not pursue the details farther. On the 5th, an opening was made into the palm of the hand, and matter was let out. In a day or two the several wounds in the finger were laid open together, so as to make one continuous incision from the point of the finger into the palm of the hand; this gave great relief by removing the excessive tension of the parts. Several other collections of matter formed in different parts of the palm, and were opened as soon as discovered.

From this time the constitutional symptoms gradually abated. The inflammation of the absorbents subsided, and the openings made into the finger and hand discharged pus freely. Poultices were constantly applied and the hand frequently fomented. The morphia was gradually diminished in quantity. His diet was improved, and tonics administered. Dr. S. was at length able to go into the country, and after an illness of six weeks he was restored to his usual health. In defiance of all treatment the flexor tendons sloughed, and consequently, when the wounds were healed, the finger remained motionless and stiff.

We have not much curtailed this case, because it is a highly interesting one. The points which deserve attention are the following:—1, There was no discoverable puncture nor abrasion of the cuticle. It would appear, therefore, that absorption took place through the skin. In one of the cases detailed by Dr. Duncan, a similar circumstance occurred. Mr. Stafford communicates another, of a similar description. In our own person, we have twice suffered from inflammation of the absorbents and violent pain, with redness and swelling in the finger, not succeeded by suppuration, but in neither instance were we aware of having suffered from either a wound or cuticular abrasion, nor was the slightest trace of any to be found. We think, then, that so far as evidence can go, it establishes the fact of contamination by a morbid poison in dissection, independently of any lesion of parts in the recipient. When we recollect the mode in which syphilis can be transmitted, we must cease to feel any surprise at this.

2. The rapidity of the progress of the symptoms in this case was striking. As Mr. Stafford observes, at four o'clock in the morning Dr. Sims first felt pain in the finger; at eight o'clock it had swelled to nearly double its usual size, and the absorbents on the fore-arm were inflamed; and at twelve the inflammation had extended to within two inches of the axilla.

3. The affection of the nervous system was remarkable also. Is that to be attributed to the action of the poison, or to the constitutional peculiarity of the individual? Whichever was the case, the effects of the muriate of morphia were most gratifying, and should be borne in mind in the management of similar cases hereafter.

4. We cordially agree with Mr. Stafford in the following remarks. They refer to a most important, because a practical point, and we would enforce in as strong terms as language can afford, the necessity of attending to it.

"This case illustrates one important point, the necessity of making an opening as early as possible into a swelling where matter is suspected to be formed. Before the openings were made, the pain was excessive, and the irritative fever was kept up by the confinement of the pus and tension of the part. As soon, however, as the matter, although in so small a quantity, was let out, the pain was relieved, and the symptoms gradually abated. It was extremely difficult for some time to ascertain whether pus was formed or not in the third phalanx, and in the different points of the palm of the hand. Sir Astley Cooper and Mr. Lawrence were both a considerable time before they could satisfy themselves on the question. At length, however, an indistinct fluctuation could be felt; an incision was made into the part, and a small quantity of pus, deep seated, escaped with the blood.

Seeing the extreme difficulty of ascertaining whether pus is formed or not, in all cases of severe injury of this description, I would recommend that an opening should be made into the suspected part where there is considerable tension, with much irritative fever, accompanied with a depressed state of the nervous and vascular system. In the first place, should the swelling contain matter, instantaneous relief would be given by its being let out; secondly, should such not be the case, the extreme tension of the part will be relieved, and also the distress occasioned by it; and lastly, the blood-vessels of the part will be unloaded."

The next case is so illustrative of the value of the plan to which the preceding quotation refers that we introduce it to the notice of our readers.

*Case* 2. In Dec. 1831, Mr. Pierce, the late assistant apothecary of the St. Marylebone Infirmary, opened the body of a woman who died from puerperal fever. He was not aware at the time that he had wounded himself, nor was there any previous wound on the hand. When he rose on the following morning, he found himself very unwell, being languid and feverish, with pain in his head. In the evening of the same day, he complained of soreness and pain in the axilla, to which leeches were applied. Fomentations and poultices, fever medicines, and aperients were employed, but the swelling increased, and spread generally to the glands and to the cellular membrane surrounding them. On the fourth day there appeared a general enlargement of the same side, extending from the third rib to about three inches above the ilium. The part gradually became more tense, and there was a deep reddish blue appearance on the surface. No fluctuation could be discovered, and no prominence at any particular part of the enlargement. The constitutional symptoms now put on a very alarming character; the pulse was 140 and fluttering, there was extreme nervous depression, anxiety and distress of countenance, dry tongue, restlessness, low delirium, and dyspnœa.

At a consultation of several physicians and surgeons, it was concluded to delay opening the swelling, as there was not complete evidence of the presence of pus. The poultices were ordered to be continued, and wine and other remedies given to allay pain and support the system. During the night all the symptoms became worse, the pulse weaker, and it was evident the patient was fast sinking. In the morning, as the only remaining chance of saving life was to open the swelling on the side, Mr. Mayo made an incision nearly an inch deep and four in length into it. No pus escaped, but it bled very freely. From that moment, however, Mr. P. was relieved, and all the symptoms abated. In two days, a profuse discharge of matter made its way out from the bottom of the wound, and its quantity was so excessive that he became greatly reduced and exhausted. He was consequently allowed wine in considerable quantity, bark, meat, porter, strong beef-tea, and gravy, and, in short, nourishment of every description that the stomach could bear, and such as circumstances required. The abscess in the side communicated with that of the axilla, beneath the pectoral muscles. The discharge continued, for nearly two months, and, at the end of three months, Mr. P. recovered.

In this case the benefits derived from the incision would seem to be distinct, and it was probably owing to that incision that Mr. Pierce's life was preserved. These cases partake more of the character of diffuse than of phlegmonous inflammation, and we all know that it is not the presence of pus which determines the propriety of incisions in the former case; they are made to relieve tension, discharge blood and serum, and to afford an issue to matter whenever it *shall* form. To apply the practice of early incisions to cases of diffuse inflammation from dissection-wounds, is merely to apply to them a recognized principle in surgery.

Three other cases are detailed by Mr. Stafford, but they present no features of sufficient importance to detain us.

The last part of his paper is occupied with the consideration of the treatment of these cases.

His suggestions are not numerous. He advises the application of the argentum nitratum across the course of the absorbents, so soon as the signs of absorption have appeared. Probably this would do no harm, and probably it would not do much good. Leeches, fomentations, poultices, the muriate of morphia for nervous symptoms if they shew themselves—sudorifics, &c. for the fever—aperients, early incisions, support when the patient is suffering from extensive suppuration: such is the category of remedies reasonably recommended by our author.

But one point requires more specific allusion. It is the employment of general bleeding. Mr. Stafford is averse to it, and we cordially agree with him in his repugnance to its use. The milder cases do not require it. The severer ones are not examples of the pure phlegmasiæ, but either there are grave nervous symptoms from the general depressing influence of the poison, or it has exerted a more local though still a specific effect, and given rise to extensive diffuse inflammation. Surely neither the one case nor the other is adapted for venesection. Analogy is against it. General bleeding has been found, by reiterated experience, to be injurious in diseases occasioned by other morbid poisons, and it has been found to be on the whole injurious in ordinary diffuse inflammation. It is true that Mr. Lawrence has written a long paper in its favour, but whether Mr. Lawrence himself continues to hold or to apply the principles he advocated in that paper, is doubtful; and, whether he does so or not, we are sure that the best surgeons in London have arrived at precisely opposite conclusions. The direct evidence against general bleeding in cases of dissection-wounds, though from the nature of the subject difficult to be got at, and far from logically conclusive, is considerable. As Mr. Stafford observes—on referring to the cases published by Dr. Duncan in the Medico-Chirurgical Transactions of Edinburgh, it will be seen that several of the patients suffering from injuries from dissection were bled; in many of these cases the patients died; they never appeared to rally after the bleeding. We dwell on this point, because we believe it to be one of great importance. Perhaps there is no point of more consequence, certainly there are few of more difficulty than to determine the inflammatory diseases that will and that will not be benefited by active depletion. It would be impossible for us at present to examine a subject so comprehensive and so intricate, but we may observe that the greater the tendency on the part of the inflammation to become diffuse, the less will it be benefited by vascular depletion, and especially by general bleeding. That tendency to diffuseness argues, of itself, impaired constitutional powers, and whether the debility be the result of other and ordinary causes, or the effect of a specific poison, would appear practically to matter little, the individual, in either case, being incapable of supporting measures of a very lowering character. Whether it be scarlet fever, small-pox, diffuse inflammation after a compound fracture, or diffuse inflammation from a dissection-wound, bloodletting, if carried to any extent, is prejudicial, and serves only to lower the powers of the system, powers which are necessary to throw off the disease or its consequences.

There is another element in the consideration of the propriety of venesection, which is alluded to by Mr. Stafford, and which should be always weighed in discussions of this nature. It is the reflection that there will be sequelæ to the morbid action in existence, and that those sequelæ will make a large demand upon the patient's strength.

In some of the phlegmasiæ we know by experience that copious depletion will arrest the inflammation, or so modify it that its consequences will be comparatively trivial. So we do not hesitate to drain a patient of blood in ordinary pleurisy, or peritonitis, or phrenitis. Suppuration is not a necessary, or, in other words, not a common consequence, and we do not therefore dread it, or provide for it. But in diffuse cellular inflammation, we know, by experience, that venesection does *not* cut the matter short, that suppuration *is* a common consequence, and, such being the fact, it is surely most unreasonable to exhaust a patient, and render him incapable of sustaining the severe and protracted struggle in prospective.

The objections which have been urged against general bleeding do not apply with the same degree of force to local bleeding. This tends, when employed with judgment, to diminish the inflammation, and to limit the suppuration. Of course, it is experience only which determines this, for, à priori, it would have been impossible to have pronounced that one species of depletion would be serviceable, and that the other would not. But we are not satisfied that even local bleeding may not be carried too far. General bleeding is injurious because it weakens the powers of life. If local bleeding is pushed to a great extent, there is no reason why it may not do the same thing. Its presumed utility is its power of unloading the vessels of the part in a greater ratio than it weakens the whole system. The object, then, must be to stop at the point at which it has effected its proper purpose—an object of difficult attainment, for which no rules can be given.

If we sum up the leading principles of treatment in these severe and interesting cases, they may probably be comprised in the following axioms —local depletion—early incisions—regulation of the secretions—sedatives when nervous symptoms are severe—support when the powers of life are failing, or when they are called on for great exertions. Such are the maxims which experience, so far as it has gone, and our present amount of knowledge point out as the best for general adoption.

2. *Case of Glanders in the Human Subject. By James Johnstone, M.D. of Birmingham.**

That diseases originating in lower animals may be communicated to man, is a proposition which cannot admit of doubt. Not to mention other, less positive instances, hydrophobia and cow-pox are familiar and unobjectionable ones. Of late years, several cases have been recorded, in which glanders has appeared to have been introduced into the human subject, either by direct inoculation, or in some less obvious manner.

It must be owned, that all the recorded cases have not been satisfactory, and that not only has the evidence of inoculation been occasionally inconclusive, but the morbid phenomena themselves have been too various to admit of any accurate generalisation at present. We are reduced, therefore, to fall back on observation, and to accumulate as many facts as possible.

Dr. Johnstone, one of the ornaments of the Provincial Association, has reported such a fact in its Transactions. We shall present an account of it.

*Case.* W. W. æt. 40, a groom, of temperate habits, was admitted into the Birmingham Hospital, on the 12th of July, 1836.

He complained of severe gnawing pain in the right knee, which was very tender; and small red lines, which seemed to follow the tracks of the absorbents, were visible on the inside of the thigh; but the part was not swollen. The right elbow was slightly swollen, and the skin had a deep pink colour; but he suffered little pain in this joint except when pressed. The appetite varied; and he was more thirsty than usual. Bowels regular; urine plentiful; tongue clean and moist at the apex, but very foul at the root. He said that he had no headache, but the pupils of the eyes were much dilated, and the countenance was expressive of anxiety. Pulse 120, small, and *weak*. The conjunctiva was slightly yellow, and the skin cool, except around the right elbow.

On the 14th, the swelling of the elbow, which had been fomented, had nearly subsided, but the knee was more tender and inflamed. For several days, no marked alteration occurred, but the strength obviously declined, he grew delirious at night, the left knee became slightly inflamed on the 21st, on the 23rd the inspirations were difficult, and at 10 p. m. of that day he expired.

Such was the case at the time of admission, and from that period to the patient's death. The following is the history which he gave, and which we have purposely introduced here that it might not interfere with the narrative of the facts which were actually observed.

---

* Provincial Transactions.

He stated that in March, 1835, he was engaged in cutting up a horse, which had died of the glanders; and having, two or three days previously, been pricked by a thorn in the palm of the left hand, between the index finger and the thumb, and the wound being not quite healed at the time of the dissection, he supposed that matter from the dead horse must have come in contact with it, for on the following day his hand became swollen and inflamed, and was excessively painful. It continued in this state for five weeks, without much constitutional disturbance, leeches and poultices being alternately applied; after which the pain, swelling and inflammation suddenly receded from the hand, and made their appearance between the elbow and the shoulder. He did not remember that there was any redness extending along the arm from the hand. In nine days the inflammatory appearances vanished from this place, and the calf of the right leg immediately became affected, and continued so till the month of October. The left knee had also been affected, and still bore the marks of cicatrized sores. He was admitted into the Hospital in July, 1835, but, by his own desire, left it at the end of three weeks, without having received any relief.

Despairing of a cure by ordinary means, he endeavoured to make an open sore by pricking the part affected with a needle, in the hope that a healthy action might be thus induced; but he could never excite a proper discharge, " it was always a watery corruption."

Nevertheless, he apparently recovered in October, and returned to his work; but in January, 1836, he was attacked in the right shoulder, though much less severely than on any former occasion. He again recovered in nine or ten days, and was free from pain till the end of May, when he injured his ancle, and pain, redness, and swelling, similar to that from which he had before suffered, again made their appearance in the part. In six or seven weeks the malady suddenly left the ancle, and the right knee became affected; and in the course of a fortnight, the right elbow was also attacked. He had for some time past been occasionally subject to rheumatic pain in the joints, especially in those of the upper extremities; but he declared that he had never had syphilis, and had never been salivated. About three weeks before Dr. J. saw him, he had a severe headache, for which leeches were applied to the temples.

*Dissection.* The body was muscular. No mark of syphilis could be discovered. On reflecting the integuments which cover the upper portion of the right tibia, two abscesses were discovered, the walls of which were formed by the internal surface of the fascia, and the external surface of the periosteum. These membranes were more vascular than usual, and the latter was considerably thickened, and easily detached from the bone.

On opening the head, considerable effusion was found under the arachnoid membrane in every portion of the brain, especially at the upper part of the hemispheres. The medullary substance had a darker colour than usual, but was firm, and appeared to be otherwise healthy.

The passage of the larynx was almost closed by œdematous swelling of the glottis, so that water poured into the orifice very slowly passed through it; and the mucous membrane was much distended by effused serum. The epiglottis and trachæa were in a perfectly healthy state. The cricoid cartilage was partially ossified, and an abscess, which was in contact with the bony part, had penetrated through the mucous membrane, the texture of which was destroyed for about the space of a shilling. The lungs adhered to the sides of the chest in several places, but were quite healthy.

Dr. Johnstone admits that this case is far from offering conclusive evidence in favour of the view that it was essentially one of glanders. It was in fact an instance of cachexia, or of the indeterminate group and series of symptoms which go by that familiar name. It would be easy to select from the mercurial and syphilitic cachexiæ, which are constantly occurring, instances very similar, if not quite analogous to the preceding. The cachexiæ, no matter how induced, evince a marked tendency to affect the cellular membrane, and its modifications the fibrous and the cutaneous. The cachexiæ, too, however different in origin, when once induced are almost alike in characters and progress. So strong is this similarity, that we are usually compelled to refer for discriminating marks, to the history of the particular case, and every one who is practically conversant with medicine knows how difficult it often is to obtain one on which we can implicitly rely. We mention these circumstances, for the sake of enforcing the necessity of caution in admitting specific infections on evidence of a dubious nature.

3. *On the Poison of Measles. By R. Williams, M.D. Senior Physician to St. Thomas's Hospital, &c.**

In our last number we presented an account of the first half of the volume of Dr. Williams which treats of Morbid Poisons. In that half, the poisons of typhus and of scarlet fever were considered. In the remaining half the poisons of measles, of small-pox, of hooping-cough, and of erysipelas are treated of. We shall notice these more or less cursorily, because very much of what is stated by the author must be, ex necessitate, elementary. The poison of measles is first examined.

Measles are both contagious and infectious—that is, they appear in an individual after exposure to an atmosphere or to fomites contaminated by another individual. Measles also are often epidemic, and then the cause is supposed to be in the air. Of course we know nothing positive on this head. The amount of our information extends only to this, that at certain times measles are extremely prevalent, and that individuals become affected by them independently of any appreciable exposure to air or fomites specifically contaminated by other individuals. Such is the statement of the fact. It is put more hypothetically by Dr. Williams thus:—*that*, it seems to be a law of poisons, generally, that they vary greatly either in quantity or intensity; and the measles have been constantly observed to reign epidemically rather than sporadically, breaking out in certain years at the beginning of winter, increasing till the vernal equinox, and dying away towards the summer solstice.

The infectious nature of measles has been so often seen, that it is, and, perhaps, may be, taken for granted.

Its contagious properties were proved directly by Dr. Home, who, in 1750, inoculated for the measles. He inoculated with measly blood. The running at the eyes was very considerable, the cough was scarcely present in the disease produced in this way. The contagious nature, says Dr.

* Elements of Medicine. Vol. 1. On Morbid Poisons.

Williams, of the serum contained in the vesicles, which sometimes accompany the eruption of measles, has been proved by Mr. Wachsel. This gentleman inoculated a lad, Richard Brooks, with the fluid taken from those vesicles, and also from the vaccine pock. Both diseases were produced, the vaccine virus taking the precedence, owing perhaps to its having a shorter period of latency than the morbillous poison.

The experiments of Home have been repeated by Vogel, Brown, Monro, and Tissot; and the result has led them to imagine that a mitigated and mild disease followed. On the contrary, however, the similar experiments made by Cullen, Girtanner, Rosenstein, and Vaidy, were not attended by any such mitigation of the symptoms as in their opinions warranted the continuance of the practice, and consequently of late years the inoculating for measles has been discontinued. As late, however, as 1822, Professor Speranza, in an epidemic that prevailed at Mantua, inoculated himself and six boys, after the manner recommended by Home: they all took the disease, and it ran a mild and regular course.

Dr. Williams shews, in succession, that the disease may be conveyed by fomites—that the susceptibility to the disease, though usually exhausted by one attack, is not always so—that it may co-exist with small-pox, with cow-pox, and with hooping-cough—and he supposes that the poison of measles is absorbed and infects the blood.

In speaking of the period at which the contagion of measles is first generated, a period far from determined, he makes the following observations, which we extract.

"Many believe the poison is not secreted till after the appearance of the eruption, while others believe it takes place during the primary fever. The following cases seem to establish the latter hypothesis. A merchant set out from London to Pyrmont on the 30th of June, 1825, taking with him his wife and three children, but leaving behind a fourth child, apparently attacked with severe catarrh, and which afterwards proved to be the measles. The party arrived at Pyrmont on the 8th of July, and on the day following, one of the children at Pyrmont, the playfellow of the one left behind, was taken ill of what appeared to be a common catarrh, but as the account of the child in London having the measles had not yet been received, he was permitted to associate with the rest of the family till the 11th of July. On that day, however, the two remaining children were removed to a different apartment in the same house, while three little girls, the daughters of the host, were removed to a different part of the town altogether. On the 12th of July the eruption broke out in the child affected with catarrh, and on the 24th and 25th the five children, who had not seen the infected child since the 11th of July, or the day before the eruption, fell ill of the disease. These instances are extremely remarkable, for there was not a case of measles at Pyrmont at the time, and from the little patients being strictly secluded, the disease did not spread to any other family in the town. The infection, therefore, must have been communicated during the primary fever, and before the appearance of the eruption." 173.

Passing over the description of the varieties of measles, and their diagnosis, in which we see little to detain us, we may pause at the prognosis. The mortality from measles, observes Dr. Williams, greatly varies in different years. Percival says, that out of 3807 cases, ninety-one died, or one in forty. Watson says, that in one year at the London Foundling Hospital, one in ten died, and in another, one in three. In the same establishment, also, in 1794, out of twenty-eight cases none died; in 1793, out of sixty-nine cases six died; in 1800, out of sixty-six, four died; and the aggregate of these data, taken collectively, will give a proportion of deaths to recoveries of about one in fifteen, which nearly approximates to the calculation of Home, who estimated them at one in twelve. We quote the following passage for the benefit of our less experienced readers.

"The longer the preparatory symptoms, and the more violent they are, by so much the less mild will the distemper prove.

Spasms, or convulsions preceding the eruptions, especially in a child labouring under dentition, 'magnum periculum portendunt.' (Frank, p. 252.)

The eruptive stage of the measles is not attended with much danger either to infants or adults; but should it assume the colour of the morbilli nigri, or be intermingled with petechiæ, or ecchymoses, or should the eruption suddenly disappear, the prognosis is most unfavourable.

Diarrhœa continuing so long that the patient becomes pale and exhausted, is always unfavourable; a moderate diarrhœa is of little moment, and occurring about the tenth day it is salutary.

A moderate hemorrhage from the nose or uterus is by no means hurtful. (Frank, p. 252.)

Adults, as well as children, have in some cases hectical paroxysms twice in the twenty-four hours, without any cough or diarrhœa, and during the interval there is great restlessness, panting, and a quick, irregular pulse. The patients, when thus affected for two or three successive weeks, generally sink under the complaint. The fatal termination seems, however, to be avoided by the appearance of boils, pustules, or suppurating tubercles of the skin. It is also alleviated by suppuration of the meatus auditorius, or of the lymphatic glands.

The danger of the measles is imminent when the secondary actions of the poison fall with any degree of severity on the lungs.

The favourable circumstances are, long protracted sleep, and gentle perspiration; also, if the cough be accompanied by healthy sputa, and the urine deposits a healthy sediment.

The measles are far less dangerous to pregnant women than either the scarlet fever or small-pox. 'I have attended several,' says Heberden, 'who were greatly harassed by the violence of all the usual symptoms, but I never knew them make one woman miscarry, or to be in more danger on account of pregnancy.'

The measles are more fatal in the winter than in the spring, the summer, or the autumn.

The co-existence of measles with hooping-cough, or of measles with small-pox, is most commonly fatal, especially in winter." 183.

The treatment of measles occupies, of course, the attention of our author, and some pages of his book. But with the exception of a somewhat curious notion, we observe nothing to detain us. The notion to which we have alluded is the following. Speaking of antimony, many practitioners, he says, are in the habit of prescribing it in preference to ipecacuanha, "but as antimony has a tendency to act on the lungs, and to produce, as far as my observation has gone, mortification of their substances, it certainly is prudent to forego the use of a medicine which must predispose those organs to the action of the poison." My Uncle Toby would call this rather "hobbyhorsical," and my Uncle Toby would probably be not very far from the truth.

The section on the poison of small-pox is judicious—that on the poison of chicken-pox brief—and neither demands more particular attention from us. But the one which follows, bold in its title, bolder in its doctrines, may claim more special notice.

4. *On the Poison of Erysipelas.*

It is scarcely necessary to inform our readers, that the contagiousness of erysipelas is a matter on which physicians and surgeons are neither unanimous nor decided. The cases which have been published by Dr. Wells and others, and those which every now and then occur in private and in public practice, have proved, so far as any thing of the sort is proven, that erysipelas may be contagious. But the general experience of medical men, the facts which are continually occurring, and reasoning, so far as we can place reliance on it, combine to prove also that under ordinary circumstances erysipelas is *not* contagious, and is *not* the result of a specific poison.

But Dr. Williams is not inclined to temporise. He treats the complaint, without reservation, as infectious and contagious, and no where does he hint that it *may* be neither. In speaking of it, he talks of the poison of erysipelas —in treating of it, he *jams* it in between small-pox and hooping-cough—in short, no one who reads his chapter can avoid concluding that the author ranks it with the most undoubted morbid poisons.

As we believe this calculated to convey an imperfect, if not an erroneous, view of the disorder, and as truth on such a subject must be highly desirable, if not important, we shall examine the arguments of Dr. Williams, and endeavour to determine their real value. We shall first adduce the reasons of our author for considering erysipelas as a morbid poison.

A. "The remote cause of erysipelas," says Dr. Williams, "must exist at all times in the atmosphere, or else the human body must possess the power, when acted on by certain predisposing causes, of spontaneously generating this peculiar poison. The doctrine, however, of the spontaneous generation of any poison is ill supported by argument, and not generally received, and consequently the more probable hypothesis of the remote cause of this disease is, that a poison producing erysipelas exists at all times diffused through the atmosphere, varying greatly in quantity or intensity; for, although the disease is at all times sporadic, yet in some years it is epidemic. The occasional increased prevalence of this disease has been observed by many persons. 'There are some years,' says Mr. Calmiel, 'when among the insane erysipelas is almost indefinitely multiplied, so that it is necessary to suspend all treatment by counter-irritants, which form the basis of the cure of the insane; for the application of a seton, of a moxa, of a blister, or a slight blow, the opening of a vein, or the application of leeches, is certainly followed by erysipelatous inflammation. This year (1828),' he adds, 'has been remarkable in this respect, and the infirmaries have been literally encumbered with insane patients labouring under erysipelas.' Mr. Velpeau, in the year 1831, witnessed in the Hôpital de la Pitie, a similar epidemic prevalence of this disease, for in both the medical and surgical wards, the application of leeches, a slight operation, or even a puncture, brought on this inflammation, with all its consequences. Blache and Chomel also speak of having many times seen it epidemic, especially in the autumn of 1818, a year of excessive heat, and long drought. Erysipelas, therefore, is occasionally epidemic; it is also more frequent in summer than at any other season." 260.

If prevalence of a disease at particular times is to be deemed evidence of a special poison existing in the atmosphere, then we must have a considerable number of poisons extant. There are numerous disorders which are epidemic, or which may become so. Catarrh, sore throat, bowel complaint, the minor exanthemata, boils and carbuncles, many, in fact, of the affections of the tegumentary membranes, whether mucous or cutaneous. And do all these depend on poisons floating in the air? Or are they not more probably modes of action excited in the economy by variations in the condition of the atmosphere, independently of any poisonous matter in it?

It is obvious that there are not any means of determining this question in a positive manner. The utmost we can attain is a strong probability, the expression of minor cumulative probabilities. Analogy is almost the only method which we can apply to the solution of the case.

We conclude that catarrh, diarrhœa, and so forth, occurring epidemically at certain seasons, depend on variations in the barometrical and thermometrical conditions of the air, because they coincide, in a greater or a less degree, with the variations in question. That is the reason for the opinions commonly entertained upon the subject, and it is evidently only conjectural. It is *easier* to suppose that this is true, than to believe that a poison is developed.

Now erysipelas follows a nearly similar law. It is usually (nine times out of ten), most prevalent in variable seasons, or in extreme seasons, but particularly in the former. We look for it in Spring and Autumn—at the breaking up of a frost—in very hot Summers—during the prevalence of cold winds. We find it usually concur with other affections the consequences of such causes—with catarrh—with sore throats—with bowel complaints. Is it more natural, more easy, to imagine, that atmospheric variations should give rise to affections which evince a disposition to occur simultaneously with them, or to suppose that a more occult alteration is effected in the air, and that a poison is developed or rendered active in it? For our own parts we lean to the simpler explanation, and are disposed to disbelieve these mysterious agencies, unless the evidence of their existence is strong.

It may be said that measles, and small-pox, and scarlet-fever, affect the epidemic character. No doubt they do so. But it is not because they are occasionally epidemic that we believe them to constitute morbid poisons, but because they are proved in other ways to be such. Epidemic tendency is not an essential but an accidental character of morbid poisons. If one exhibits it, it does not follow that another shall, and neither is its absence or its presence evidence of the absence or the presence of a poison.

### B. *Infectiousness of Erysipelas.*

"The infectious nature of erysipelas is a doctrine of modern origin, and although not universally received, yet the facts in support of it are so many and so irrefragable, that no doubt can be reasonably entertained of its truth. Among the many proofs of this law are the following:—in the year 1760 a person labouring under erysipelas was brought into St. Thomas's Hospital, and shortly after died. From some accident, a patient suffering from a different disease was put into the same bed, but without the usual precaution of airing the mattress, and changing the bed-clothes, and this man was also seized with erysipelas of the face, and he died. Several other patients, also, were subsequently attacked with this disease in the same ward, together with the sister, and she died. At length this disease acquired so formidable a character that a report got abroad the plague was in the hospital, which was only silenced by the physicians and surgeons contradicting it by a public advertisement. A knowledge of this fact directed the attention of the late Dr. Wells, of St. Thomas's Hospital, to the infectious nature of erysipelas, and he has related in the second volume of the Transactions for the 'Improvement of Medical and Chirurgical Knowledge' several striking cases of the communication of the disease, either by infection or by direct personal contact, that fell under his own observation. These facts have been corroborated by additional instances observed by Dr. Pitcairne, also by Dr. Baillie, who, in the years 1795 and 1796, saw it spread in St. George's Hospital, and by Dr. Cullen, who had seen the like circumstance in the infirmary at Edinburgh. From that time evidence to a considerable amount has gone on accumulating, so that little doubt remains in the minds of the great majority of the profession in this country, that erysipelas is both an infectious and contagious disease. In St. Thomas's Hospital, where many opportunities have presented themselves for studying the laws of this disease, there is, I believe, no physician or surgeon not fully persuaded of this fact. For since the year 1829 no less than four or five of the wards of that establishment have been cleared out, whitewashed, and painted, in order to stop the wide and fatal spread of this disease. The infectious nature of erysipelas has, indeed, on many occasions, appeared so manifest, and the danger often so imminent, that the medical officers considered it an imperative duty to recommend to the governors the necessity of sacrificing the houses on the north side of St. Thomas's Street for the purpose of procuring a more complete ventilation. This recommendation has, in all probability, led to the rebuilding of the new wards of this establishment, which combine, in so remarkable a degree, space and ventilation with warmth and comfort, so that it is hardly possible to suggest any improvement in their construction. A month, however, seldom elapses without some new and striking instance of the infectious nature of erysipelas occurring in the older parts of the building, and it is to be regretted that it has even spread this year in the new wings, for six persons in Anne's Ward have been seized with this disease subsequently to the admission of two or three erysipelatous patients into the ward. It has been said that this circumstance can be explained on the ground that erysipelas was prevalent during the last summer; but if we remember that in London erysipelas did not attack one person in five hundred, perhaps not one in a thousand, it will be plain that the chances against six persons being attacked out of twenty-six are enormous. We can, also, nearly to a certainty, determine the cases which will be liable to an attack, supposing erysipelas to be introduced into a ward; for the convalescent from fever, the swollen with dropsy, the syphilitic, and the scrofulous patient, are its surest victims. Of the six persons who have been mentioned as seized with erysipelas in Anne's Ward, one had ovarian dropsy, two ascites with albuminous urine, one was in the crisis of a dangerous fever, one had scrofulous glands of the neck, and the sixth only, one of the nurses, was a young and healthy person. It is remarkable that phthisical patients, however debilitated, are seldom liable to this disease. It is apprehended that the proof of the infectious nature of erysipelas after these facts is strictly demonstrated." 263.

In a pendent to this passage, Dr. Williams endeavours to decide the distance to which the infection of erysipelas may be carried. He admits it is difficult to determine such a point, but he offers some data for an opinion on it. For example, in a case which occurred under Dr. Roots, in King's Ward, the patient lay from the infected source at least fifteen feet. But the greater space of the new wards has not been found a sufficient protection against the wide spread of this poison. The breadth of the old wards is nineteen feet, while that of the new is twenty-eight feet; still it has spread in both cases, not merely from bed to bed, but to the opposite sides of the ward, and even to patients lying three or four beds off on that side. The miasmata of typhus rarely spread, it has been stated, from bed to bed, and, consequently, not more than from three to four feet around the patient's person; but the miasmata of erysipelas spread from twenty to thirty feet from the infected source, and, consequently, this disease is infinitely more infectious than typhus. Such are the deductions which would seem to spring from the premises of Dr. Williams, supposing them to be correct.

But there are some obvious objections to the whole chain of reasoning. It has been admitted that erysipelas is peculiarly prone to assume an epidemic character. When it thus prevails, the inmates of an hospital would suffer from it, of course, as well as those who are out of hospitals. If several persons in one ward are affected, how are we to determine that they are suffering from a specific infection, and not from the general epidemic influence?

Dr. Williams endeavours to answer this objection by the numerical method. He replies that, in the whole of London, erysipelas did not attack one person in five hundred, while, in St. Thomas's Hospital, six out of twenty-six were seized. This difference of ratio he ascribes to the agency of infection in the latter instance.

If all persons, those in and those out of hospitals, were equally prone to contract the disease, this mode of argument would be satisfactory. But that is not the fact. Dr. Williams admits that it is not the fact, for he observes that we can nearly to a certainty determine the cases which will be liable to an attack—the convalescent from fever, the dropsical, the syphilitic, and the scrofulous. In other words, erysipelas is a disease which peculiarly requires for its development, predisposition on the part of the individual. The efficient causes of such predisposition are concentrated to a high degree in hospitals, and, therefore, when erysipelas prevails, it prevails most extensively in them. It is clear that Dr. Williams's application of numbers is, on his own shewing, a fallacy. We pass to the contagiousness of erysipelas.

c. "*Contagious:*—The contagious nature of this disease has been proved by Dr. Willan, who affirms, if a person be inoculated with the fluid contained in the phlyctenæ of a genuine erysipelas, that a red, painful, diffuse swelling, analogous to that from which the fluid was derived, is produced.

*Fomites.*—The case given by Dr. Wells of the patient who caught erysipelas in consequence of being laid in the unchanged bed of one that had died of this disorder, and the difficulty that has been experienced in eradicating this disease from the wards of St. Thomas's Hospital, are strong proofs of this law. In the navy, also, we learn from the second volume of Mr. Travers's book on constitutional irritation, that the contagious nature of erysipelas, and consequently its communication by fomites, is so generally admitted, that it has become a debated question, whether its ravages are best limited by swabbing the decks in the usual manner, or by dry-rubbing them. Among the latest evidence of the disease being communicated by fomites is that of Dr. Gibson of the Montrose Infirmary. 'Some days,' says this physician, 'after the admission of a female patient into the Montrose Infirmary with abscess of the hand and caries of the bones of one finger, the patients in the two next beds to her were seized with erysipelas, and, on enquiry, it was ascertained that the suppuration of the woman's hand had been caused by an attack of erysipelas.' The patients were all now removed from that ward, which was cleaned, whitewashed, and fumigated. 'Yet when the patients were again placed in that ward the disease again made its appearance, and it was found necessary to remove the whole of the patients from our little infirmary, and to take every precaution before the contagion was eradicated.' " 264.

Positive evidence and direct facts have apparently established both the infectious and contagious nature of erysipelas. That evidence and those facts, when marshalled by a friendly hand, are most imposing, and they tend to shew that erysipelas is a *most* infectious and a *most* contagious malady. The inexperienced medical practitioner, who should peruse Dr. Williams' pages, would rise, we are sure, with that conviction, and would enter on practice with the strongest apprehensions of a complaint depicted in such frightful colours.

If such a practitioner were told *that* a large proportion of physicians and surgeons were sceptical of these infectious and contagious properties, *that* no precautions at all commensurate with the occasion were taken to prevent their necessary consequences, and *that*, notwithstanding this supineness, erysipelas was *not* found to spread in private or in public to any thing like the extent which would seem inevitable, he would either wonder at the obstinacy of mankind in rejecting the evidence of their very senses, or he might suspect that Dr. Williams had not laid before him the *whole* truth, and had leaned exclusively on such particulars as suited his hypothetical views. And probably this suspicion would prove to be well founded.

The objections which we would take to the general tone of Dr. Williams' inferences are the following:—

*a.* As we before observed, the facts that he has adduced in favour of the infectiousness and contagiousness of erysipelas, prove, if they are to be received as representing its ordinary characters, that it is a *very* infectious and contagious malady. To this we apprehend that general experience is opposed. The common opinion founded on the extensive basis of common observation regards appreciable infection and contagion in this complaint as the exception rather than the rule.

*b.* The predisposing causes of erysipelas are such as do not operate to any thing like the same extent with the other *admitted* morbid poisons. A person must be usually debilitated before he can receive it, or rather his system must be brought to that condition which is not represented by pure debility, and which, to save our entering on tiresome particulars, we shall shortly and loosely call " a bad or irritable habit of body." The term is familiar, and the complex idea which it represents is familiar too. Now this is certainly not *so* true of small-pox, measles, scarlet fever, cow-pox, hooping-cough, &c. We leave fever out of the category, because that is a vexata quæstio.

*c.* The exciting causes are such as we do not see occasion any other complaint the effect of a specific poison, and such as do occasion other complaints which are not the effects of any poison at all. Cold is a common cause. We know a lady who has erysipelas of the face whenever strong north-easterly winds set in. We have already stated that the same sort of weather which occasions sore throats and catarrhs, occasions also erysipelas. Atmospheric vicissitudes, in short, are a fruitful source of erysipelas. Dr. Williams has alluded to the doubt that prevails respecting the superiority of dry-rubbing over swabbing on board-ship, during the prevalence of erysipelas, or as a preventive of it. But he has not ventured to tell us how dry-rubbing, which has won the day, should destroy infectious matters better than washing. The fact is, that swabbing has been denounced, because it was believed that the *cold and wet* gave rise to the complaint, not because it was not calculated to destroy fomites. Certain articles of diet notoriously occasion erysipelas. Shell-fish and salmon frequently do it, and in certain individuals they always do it. Acids have been known to produce it. Is any thing analogous to this seen in true infectious diseases? The most fruitful causes of erysipelas are wounds. We cut out a tumor, or we make an incision, and erysipelas follows. Do we see this in small-pox, or measles? Nay, the kind of erysipelas is modified by the kind of wound. A mere prick or scratch is usually followed by cutaneous erysipelas—a deeper wound involving the cellular tissue by diffuse inflammation of that tissue, or by that tertium quid between cutaneous erysipelas and diffuse cellular inflammation, erysipelas phlegmonodes—a clean cut is less likely to be succeeded by erysipelas than a punctured wound is—a punctured wound is less likely to induce it than a lacerated one. Is any thing similar to this observed in the true morbid poisons?

*d.* An attack of erysipelas does not exhaust the susceptibility of the individual. He is just as likely, nay, he is more likely to suffer from it again. Here is another general distinction between erysipelas and the recognized morbid poisons.

*e.* A candid consideration of all the circumstances connected with this interesting and important subject, must lead us, we apprehend, to adopt one of two conclusions. Either we must concede the morbid poison of erysipelas to be of a very dissimilar character to that of the other morbid poisons, and place it in a niche by itself—or, we must suppose it to be merely a mode of inflammatory action,* requiring a particular constitutional state for its development, and called into operation by very various and opposite stimuli. In this view, a person in that condition prone to erysipelas would have it excited by cold, by articles of diet, by injuries, by contact with another individual labouring under it, by fomites, by infection, perhaps by other less obvious excitants. This doctrine appears to us to be most consistent with the facts, and most easily reconciled in the present condition of our knowledge with the evidence and the phenomena. It has the merit of assuming as little as possible, much less than the doctrine opposed to it, as little indeed as is assumed in any hypothesis in medicine.

We do not think it necessary to pursue Dr. Williams farther through the subject of erysipelas, as, in a late number of this Journal, we noticed very fully his views respecting its treatment. Our readers may remember that he places his principal dependence on wine, and that he advocates the ex-

---

* We admit that the term "mode of action" cannot be received as a true distinctive character, as we cannot determine that the phenomena which follow inoculation with a morbid poison are not themselves a mere mode of action; indeed it is most likely that they are so. But we use the expression for want of a better, and we only mean to imply by it, a quantitative rather than an essential difference—a more obvious mode of action in lieu of a more recondite one. It is to be regretted that our physiological knowledge is too imperfect to enable us to avoid these loose expressions and uncertain reasonings. They are, perhaps, so inherent in the nature of the subject, that all medical hypotheses must be more or less vague and metaphysical in their enunciation and their proofs. —*Eds.*

hibition of it in a very strong, if not in rather an indiscriminating manner. But we need not revive a question on which we have previously dwelt at some length.

The chapter on Pertussis is a sensible compendium of opinions and practice. It requires no further attention. But the last chapter, which is a curious tail to the morbid poisons, and is dedicated to the bromide of potassium, may give us a short pause.

### III. On the Uses of the Bromide of Potassium in Diseases of the Spleen.

It is to simple enlargement of the spleen, such enlargement as ensues from remittent or intermittent fever, that Dr. Williams limits his remarks. He first shews, or attempts to shew, that mercury is not of service to it—he then complains that no ordinary remedies relieve it—and he finally introduces the bromide of potassium with the following bow.

"It has appeared, therefore, that a more efficient remedy for these forms of disease was a desideratum in medicine; and after the success which attended the exhibition of the iodide of potassium in some forms of secondary syphilis, it seemed reasonable to expect that bromium might afford salts, possessing similar, or at least equally valuable medicinal properties; especially as that elementary fluid is remarkable for its extremely pungent and acrid qualities. With this object, therefore, I was induced to institute a treatment by the bromide of potassium in many different diseases; but as a general result, I have not been able to attribute to that substance any other valuable medical property than that of reducing enlarged spleens; a property which, if established by further experience, would render it invaluable in our colonies, diseases of that viscus and their concomitant symptoms being the endemic cachexia of tropical countries. Diseases of the spleen are not common in London, even in the practice of a large hospital; and the following cases are all that I have had an opportunity of treating with this medicine; but as it was singularly successful in all, there is a high probability that its powers over this disease may be of a specific character; and being anxious that the experiment should be repeated by those who have more extended opportunities than myself, I have submitted them thus early to the public." 336.

Dr. Williams relates four cases in corroboration of these statements. Of the four we shall give the particulars of one, incomparably the most flattering to the remedy. This we do by way of encouraging our readers to test its powers in such cases as may seem adapted for it.

*Case.* Thomas Nursay, aged fourteen years, was admitted into St. Thomas's Hospital, September 13, 1833. He had been ill eighteen months, and had taken a variety of medicines; but his friends could not trace his present disorders to any known source, nor did they admit that he had at any time been exposed to marsh miasmata. On examining his abdomen, both the liver and the spleen were found enormously enlarged, so that they met at the median line, and could be traced downwards till they were lost in the pelvis. Their edge, also, was hard, and their substance solid and unyielding, while much fluid was detected in the abdominal cavity; the boy's countenance was sallow and emaciated, his legs dropsical, his belly unsightly and protuberant, and the prognosis from all these circumstances was most unfavourable. Dr. Williams tried, at first, the supertartrate of potass, then the iodate of potass, and then the ioduret of mercury, by which the patient was salivated. The improvement not being satisfactory, Dr. W. prescribed, on the 13th of May, 1834, potassii bromidi, gr. 1, ter die, and gradually increased the dose to gr. iv., ter die ex misturâ camphoræ. He continued taking the medicine until the 10th of July, when he became slightly jaundiced.

"As I was apprehensive the bromide of potassium might have caused this derangement of the liver, which I was satisfied would quickly subside under a treatment by the sulphate of magnesia, I prescribed that remedy is ʒss. doses, and for a time omitted the bromide of potassium. By the 11th of August the jaundice had disappeared, but no diminution of the diseased viscera appeared to have taken place since he had ceased to take the bromide of potassium. The sulphate of magnesia was therefore omitted on the 11th of August, when the bromide of potassium, grs. iv., and which was increased on the 15th to grs. v., was again prescribed, and continued with only an occasional intermission till the 7th of October, 1835, or for a period of about fourteen months. A few weeks after he began this long course, he was able to leave his bed, and to be about the ward, and his recovery was now so rapid, that for many months subsequently he amused himself in spreading plaisters, and making himself generally useful in the hospital. At the time of his discharge, the liver and spleen, as far as could be judged, were not more than one-third their former size, while their previously hard edge and consistency could no longer be felt. As the general health of the boy was now good, and he had grown many inches, his friends at the hospital provided him with a place as foot-boy, so that I saw him occasionally, and in good health, for many weeks after his dismission. He was, however, at length sent away for some misconduct, and I have not since heard of him." 338.

In the next case, much benefit appeared, on two occasions, to have been derived from the use of the bromide, but she was twice attacked with hæmorrhage from the stomach and bowels, and the second seizure proved fatal. On dissection, the spleen was found to be thrice its natural size, but healthy in its structure. The peritoneum was greatly thickened and diseased, and the liver was knobbed, and of the colour of pale dried Turkey rhubarb. The patient did not therefore die of simple splenic disease.

In the next case, the patient, who was admitted on the 16th of October, 1834, and was discharged on the 2d of April, 1835, calculated that the spleen was reduced in size two-thirds by the bromide.

In the fourth case, both the spleen and liver were felt, on the admission of the patient, to be enlarged and hard. On her dismissal, three months afterwards, neither spleen nor liver was distinguishable.

Actual experiment will go farther to determine the real value of the bromide of potass in cases of enlarged spleen, than a whole volume of reasoning. There is this good feature in the remedy, that it seems to do no harm.

We now return to the Provincial Transactions.

One hundred pages are occupied with a Medico-Topographical, Geological, and Statistical Sketch of Bolton and its neighbourhood, by Dr. James Black, of the Royal College of Physicians of London. The sketch in question is highly creditable to the zeal, the information, and the judgment of its author, and will repay perusal in the original. It is not adapted for analysis, and we must content ourselves with this barren mention of it.

Nor can we do more than refer to an interesting paper on the Unity of Organic Structure, by Dr. Dick, of Clifton, a man of philosophical tastes, and of a cultivated mind. The subject has been too lately discussed in this Journal, to permit us to resuscitate it for the present.

Some Observations on the Influence of Sleep on Digestion and Secretion, by Dr. Wakefield Scott, Physician to the Liverpool South Dispensary, are judicious, but too general for our pages.

### IV. CASE IN WHICH THE MEMORY OF LANGUAGE WAS LOST. By T. SHAPTER, M.D. Physician to the Exeter Dispensary, and Lying-in-Charity.

Several cases have been recorded, in which the memory or the recollection of language was partially or wholly lost.* As such cases are too common to be considered matters of mere curiosity, yet too rare to be familiar, and as they bear on a subject with which medical men generally are too little acquainted, the philosophy of the human mind, we shall present an account of Dr. Shapter's own case, as well as of some others which he quotes.

Referring to Louyer Willermay's article in the Dictionnaire des Sciences Medicales, Dr. Shapter quotes his classification of the diseases of memory, as a function. He divides them into—1, *Enfeebling*, incomplete, or complete; 2, *Destruction*, partial, or confirmed.

The diseases referable to the first class, are for the most part caused by congenital malformations or the advance of old age: while those of the second class have their origin occasionally in acute disease, though more frequently in cerebral lesions.

Dr. Abercrombie observes, that it is chiefly in attacks of an apoplectic nature that we meet with examples of loss of memory on particular topics, or extending only to a particular period. It is true that such partial loss of memory is most commonly the result of such causes as are calculated to produce compression of the brain, but it occasionally follows what is considered as concussion. After that accident it is not very uncommon for the patient to have forgotten events which happened a little prior to the occurrence of the accident. Sir B. Brodie, for instance, mentions the case of a groom, who was cleaning a horse, and was kicked so as to produce concussion of the brain. He quickly recovered from it, and, having quite forgotten what he had been about, he informed those about him that he must "go and get the horse out of the stable to clean him."

It is nowise surprising that the memory of language should be interfered with, while that of things continues unimpaired. The mode in which we acquire our ideas of these two objects of memory is different. A man learns the form of a table, for example, through the medium of the senses—its physical qualities make a powerful and, we may say, direct impression on

* Memory, in this sense, implies the retention in the sensorium of images, whether abstract or direct—recollection, the power of voluntarily recalling them.

the sensorium. But its name is an arbitrary and an abstract subject of conception, and no fixed nor necessary relation exists between the name and thing. As the respective ideas are differently acquired, so they may be differently lost, and so, in fact, we find that they are.

We will now quote some of the cases which Dr. Shapter himself quotes.

"Dr. Abercrombie states, that a gentleman whom he attended, after recovering from an apoplectic attack, knew his friends perfectly, but could not name them. Walking one day in the street, he met a gentleman to whom he was very anxious to communicate something respecting a mutual friend; after various ineffectual attempts to make him understand whom he meant, he at last seized him by the arm and dragged him through several streets to the house of the gentleman of whom he was speaking, and pointed to the name-plate upon the door. And he quotes Dr. Gregory, as mentioning the instance of a lady, who, after an apoplectic attack, recovered correctly her ideas of things, but could not name them. In giving directions respecting family matters she was quite distinct as to what she wished to be done, but could make herself understood only by going through the house and pointing to the various articles.

Louyer Willermay refers to the instance of a man who, after an accident, could not recall the names of his relations; and of another, who could recollect no proper names without the assistance of his friends.

Dietrich (in *Archivis*) records a case of *amnesia verborum*, in which facts were remembered, but expressions were wanting to retrace and render his ideas.

In the *Ephemerides* is the case of a man, about 60 years of age, who, after an apoplexy, entirely lost the art of reading, even that which he himself had written; the power of doing which, in many languages, whether after the dictation of others, or to express that which he himself desired, was fully retained. He had also lost the art of spelling orally." 323.

This latter case is not very comprehensible. Of course, Dr. Shapter cannot mean, cannot understand that the impossibility of "reading" depended upon mere inability of articulation, for such a case would be a common one. If the inability to read be a mental impotence, and resulted from inability to comprehend what was written, that seems incompatible with being able to write, and to understand what others said. It is surely difficult to conceive that the combinations of language should be intelligible when that was oral, that the individual should be able to write them, and yet that he could not comprehend them orally. The case would appear, then, to have been simply one of impaired power of articulation. But to proceed.

"A young woman of weak intellect, subject to headaches and '*mal réglée*,' at the age of 21 experienced an attack of apoplexy. In her convalescence it was observed that she had lost all recollection of persons and occurrences. She early recollected her mother, without the power of calling her by name; at the end of a month she pronounced some words, though but very imperfectly, and her efforts to express herself involved her in almost unintelligible periphrases.

Mason Good states the instance of a man admitted into St. Thomas's Hospital with a brain fever, who, as he grew better, spoke to his attendants, but in a language they did not understand. A Welsh woman, going by accident into the ward, heard him, answered him, and conversed with him. It was then found that the patient was by birth a Welshman, but had left his native land in his youth, that he had forgotten his native dialect and used the English for the last thirty years; yet, in consequence of this fever, he had now forgotten the English tongue and suddenly recovered the Welsh.

Boerhaave gives a still more extraordinary instance of oblivion in the case of a

Spanish tragic author, who had composed many excellent pieces, but so completely lost his memory, in consequence of an acute fever, that he forgot not only the languages he had formerly learnt, but even the alphabet; and was hence under the necessity of beginning to read again. His own poems and compositions were shewn to him, but he could not be persuaded that they were his production. Afterwards, however, he began once more to compose verses, which had so striking a resemblance to his former writings, that he at length became convinced of his being the author of them.

Without adding further to the details of these anomalies, I shall conclude by offering the following references, where cases of somewhat the same complexion may be met with:—The article in the *Dict. des Sciences Medicales.*—*Journal General de Medecine.*—*Study of Physic,* by Mason Good, vol. IV.—*Centenary of Observations.*—*Dr. Abercrombie on Diseases of the Brain and Spinal Cord,* p. 176 and 214.—*Crichton on Mental Diseases,* p. 370." 324.

To these we may add one or two cases which occur to our minds at the time.

Sir Astley Cooper refers in his Surgical Lectures to a case, which is almost a fac-simile of that of the Welshman. A German sugar-baker in London, forgot, after an injury of the head, the English language which he had previously acquired, and remembered only the German.

Baron Dupuytren relates in his Clinical Lectures the case of a man, admitted, if we remember rightly, into the Hôtel Dieu, who, after an injury of the head, forgot all language except the monosyllables *pa-pa,* which he rejoined to all questions.

We saw a nearly similar case at the siege of Antwerp. A French soldier received a compound fracture of the cranium, opening the superior longitudinal sinus. There were, in the first instance, the symptoms of compression. When we saw him, in the hospital of Antwerp, he understood all that was said to him, and seemed quite intelligent. But he could only reply *ba-ba* to interrogatories. It was rather singular to observe his evident vexation at his inability to give expression to his ideas.

We will now attend to the particular case, which Dr. Shapter adds to the existing stock. It is circumstantially, and, we do not doubt, it is faithfully reported.

*Case.* Pietro Gillio, LL.D. æt. 40, a native of Italy, is, or rather was, a man possessing great comprehensiveness of mind, much vigour of intellect, of extensive acquirements, deeply read in metaphysics and general literature, and the perfect master of several languages.

In consequence of having been a prominent agent in the insurrection of Piedmont, he was condemned to death. Fortunately he effected his escape, and, since that period, has been a solitary wanderer, for some years in Spain and the Channel Islands, but latterly in England, where he supported himself by teaching the Italian and Latin languages.

Having been exposed to anxiety of mind, study, night-watchings, fastings, and cold and damp, he became affected on the night of the 14th of April, 1835, with headache, vertigo, and vomiting, succeeded by an indescribable confusion, after which these symptoms subsided.

On the 15th, Dr. Shapter was called to him, in company with Mr. Froom.

"We found him in a state of great excitement and irritability, pacing hastily up and down his chamber with unequal steps. He was incapable of articulation, and there was an almost total loss of the memory of language; for though his attention was readily attracted by speaking to him, yet the purport of what was said appeared to be in no way understood: if there were any indistinctness of hearing, it must have been but very slight. Deglutition difficult. The pupil of the right eye dilated, and but slightly answering to the impulses of light; the sight distant and indistinct; that of the left eye natural; the general expression of the eyes restless, and watching with anxious quickness those in the room. Pain in the back part of the head, but apparently not acute. Pulse rapid, unequal, 120; on the right side strong, full, and vibrating, especially pronounced in the right subclavian and carotid arteries; on the left side, the arterial action small and weak. General weakness of the left side, but not amounting to paralysis, excepting for the first hour or two after the attack. His landlady says, that at breakfast this morning he was silent, irritable in manner, and looking anxious; that suddenly he made some effort as if to speak, and then rushed hastily from the house.

The usual antiphlogistic treatment indicated was pursued, such as bleeding, blisters, and purgatives. We early found, however, that he had not stamina to permit such means to be carried to any great extent." 314.

Not to pursue details of treatment which Dr. Shapter judiciously avoids, we may cite a report dated 6th of June. The arterial action of the right side is still tumultuous in the extreme. He can recollect *portions* of a few words, and, after repeated trials, can write some of the shortest ones correctly, without the assistance of a dictionary; but words of three or four syllables are far beyond his powers of concentration; his efforts at composing a sentence are unavailing, as well as the understanding one addressed to him: he has no command of tongue. He is now studying, with the most feverish anxiety, the English lexicon, and, in great measure, manages to explain himself by pointing to particular words; but his capacity for relearning language appears limited and confined.

After this he had an excessive secretion from the membrane of the nose and fauces. In October, he complained of some tenderness on pressure over the lumbar vertebræ, which was relieved by the application of leeches and a blister. He now took to reading various books on diseases of the brain, as well as on worms, to which he said he had been prone. He occasionally drew up reports of his symptoms, and one, which he received about Christmas, is transcribed by Dr. Shapter. We may observe that, in the begining of December, he sent a memorandum in which he took a comparative view of his symptoms, stating the whole number as 100, and then giving each symptom its relative proportion according to his estimate of its intensity and importance. The following is the report previously alluded to.

" 'Sir dear—have a symptom of illness—viz.—1, spit in night and day—2, dry cough—3, a unequal pulse—4, no sleep—5, uninclination to go to stool and non-evacuate thing *quite*—6, swoon—7, loathing of food and other times a voracious appetite—8, a privation of speech—9, foot, hand bad, a hinde right—paleness of the face and times a red of the face—11, whitish colour urine (teeth, nose,—throat).

'In first attack 15 april, I had swoon in stool, not evacuate quite the *fœces;* and was sleep and was awaken and privation.

'(Mr. Duval).

'In child in is pains of worm—medicine—rue and wormwood.

'In 15 year, the same pains, medicine, oil, &c.

'in jersey—no medicine except rhubarb; in Guernsey—medicine—calomel; in Plymouth—no medicine; in Exeter is privation of speech.
'Mrs. ———— non speak true to Dr. Shapter, viz. 1, 2, 4, 5,
'(non speak,—write). *P. Gillio.*'"

In September, 1836, having received a free pardon from the King of Sardinia, and being about to return to Vico, his native place, Dr. Shapter took a final note of his condition, which we introduce.

"Has now a nearly perfect recollection of facts, of ideas, and of his past life generally; and has also recovered the recollection of many words when written before him, and to a lesser extent when spoken to him: this difference does not depend on any deafness. His powers of reading are soon exhausted; and he has, for the most part, lost the faculty of properly arranging and constructing his sentences, and is now almost totally incapable of articulating with correctness the few words he has with difficulty re-acquired. His general irritability is much decreased, and the pain on pressure of the spinal column has subsided entirely; but he complains much of painful pulsations in the posterior part of the head and neck, occurring especially during the night and towards morning. Pervigilia; pulse 104, in right side strong, left weak; the general strength of the right side restored; pupil of right eye still dilated, the sight rather more distant than that of the left; the indistinctness of vision almost recovered from; habit of body costive; appetite good only towards evening. General health from the period of the first attack, though slowly, yet progressively improves." 318.

Dr. Shapter drew up a short abstract of his case, and gave it to him with appropriate directions for his treatment. Dr. S., if he were to hazard an opinion on the nature of the case, "would refer the proximate cause of the symptoms to the bursting of a bloodvessel at the base of the brain, or the superior portion of the spinal column; and that some coagulum has been formed near that centre whence arise the glosso-pharyngeal and lingual nerves. The eye-sight, it may be recollected, was not particularly affected, but there was some loss in the powers of adaptation of the right eye; we may therefore conclude the optic nerve to have been intact, but that there was some affection of the motor nerves of these parts." It is certainly most probable, that the symptoms, in this case, depended on sanguineous extravasation. But the affection of particular nerves, as of the lingual and the motor nerves of the eye-ball, is, unfortunately, not found on all occasions to be conclusive proof of the existence of pressure at or about the *apparent* origins of those nerves. The associations of different portions of the brain by means of the commissures and of the extension of the fibres, are too complicated to permit us, in the present condition of our knowledge, to form more than a few general accurate conclusions.

We may observe that these losses of special intellectual functions after cerebral lesions, are among the most satisfactory proofs of the truth of the essential *principles* of phrenology. But whilst they go to establish its principles, they have yet contributed but little to the confirmation of its details.

We have now noticed all that requires notice in the Medico-Chirurgical and in the Provincial Transactions.* We would recommend the members

* Two or three short cases which remain will be found in the Periscope of the present number.

of the respective Associations from which they emanate to exert themselves to maintain not only their own scientific character, but that of the medical profession of Great Britain. We will always endeavour to aid such an object, and to give all the publicity in our power to those papers which are calculated to promote it.

